# Dynamic Left Intraventricular Obstruction Phenotype in Takotsubo Syndrome

**DOI:** 10.3390/jcm10153235

**Published:** 2021-07-22

**Authors:** Davide Di Vece, Angelo Silverio, Michele Bellino, Gennaro Galasso, Carmine Vecchione, Giovanni La Canna, Rodolfo Citro

**Affiliations:** 1 University Heart Center, Department of Cardiology, University Hospital Zurich, 8091 Zurich, Switzerland; davidedv@gmail.com; 2 Department of Medicine and Surgery, University Hospital of Salerno, 84131 Salerno, Italy; angelosilverio1988@gmail.com (A.S.); michelebellino8@gmail.com (M.B.); ggalasso@unisa.it (G.G.); cvecchione@unisa.it (C.V.); 3 Vascular Pathophysiology Unit, IRCCS Neuromed, Via Atinense, 18, 86077 Pozzilli, Italy; 4 IRCCS Humanitas Clinical and Research Center, Applied Diagnostic Echocardiography Unit, 20089 Rozzano, Italy; lacannagiovanni.cardio@gmail.com; 5 Cardio-Thoracic-Vascular Department, University Hospital “San Giovanni di Dio e Ruggi d’Aragona”, 84131 Salerno, Italy

**Keywords:** Takotsubo syndrome, left ventricular outflow tract obstruction, echocardiography, heart failure, cardiogenic shock

## Abstract

Takotsubo syndrome (TTS) is characterized by acute, generally transient left ventricular (LV) dysfunction. Although TTS has been long regarded as a benign condition, recent evidence showed that rate of acute complications and in-hospital mortality is comparable to that of patients with acute coronary syndrome. In particular, the prevalence of cardiogenic shock ranges between 6% and 20%. In this setting, detection of mechanisms leading to cardiogenic shock can be challenging. Besides a severely impaired systolic function, onset of LV outflow tract obstruction (LVOTO) together with mitral regurgitation related to systolic anterior motion of mitral valve leaflets can lead to hemodynamic instability. Early identification of LVOTO with echocardiography is crucial and has important implications on selection of the appropriate therapy. Application of short-acting b1-selective betablockers and prudent administration of fluids might help to resolve LVOTO. Conversely, inotrope agents may increase basal hypercontractility and worsen the intraventricular pressure gradient. To date, outcomes and management of patients with TTS complicated by LVOTO as yet has not been comprehensively investigated.

## 1. Takotsubo Syndrome: Clinical Characteristics and Outcomes

Takotsubo syndrome (TTS), also known as stress cardiomyopathy or “broken-heart syndrome”, is an emerging condition first described in Japanese women during the early 1990s. It is characterized by acute and usually transient left ventricular (LV) dysfunction [1,2]. Several triggering factors may lead to the onset of TTS. These include negative or positive emotional stressors and physical stressors due to severe medical conditions, such as end-stage chronic obstructive lung disease, intracranial hemorrhages, or post-surgical status.

The clinical presentation of TTS resembles that of acute coronary syndrome (ACS). Chest pain, dyspnea, or syncope are common symptoms at onset [3]. Several electrocardiogram (ECG) abnormalities are detected in most TTS patients [2,4,5]. A slight increase in cardiomyocyte injury biomarkers is frequently observed in TTS. Typically, the rise of serum troponin is disproportionately low compared with the wide extent of regional wall motion abnormalities [2].

In most cases, patients with TTS present normal coronary arteries. However, a co-existing coronary artery disease is observed in about 15% of cases in the absence of a culprit lesion [6]. While transthoracic echocardiography is the first-line imaging modality in patients with suspected TTS, coronary angiography including left ventriculography is currently considered essential to rule out ACS [7]. Due to the possibility of performing tissue characterization, cardiac magnetic resonance imaging (CMR) plays a central role in the assessment of patients with suspected TTS. In this sense, myocardial edema, which can be detected by T2 weighted black blood imaging, may be considered as a hallmark of TTS. The detection of myocardial edema rather than fibrosis or necrosis (with late gadolinium enhancement) is helpful to rule out different diagnoses, such as myocardial infarction or myocarditis [8,9].

**Figure 1:**
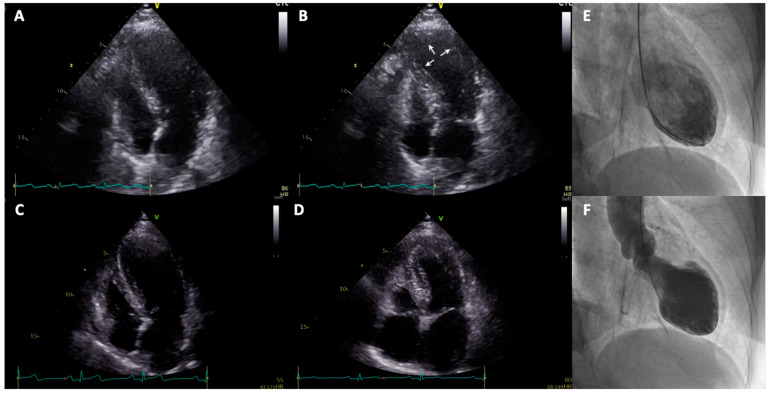
Apical 4-chamber view: in telediastole (**A**), in telesystole (**B**) showing apical ballooning (see arrows) in TTS on day 1; in telediastole (**C**), in telesystole (**D**) showing normal apical contractility on day 6. Left ventriculography (RAO 30°) in telediastole (**E**), in telesystole (**F**) on day 1 showing apical ballooning and hypercontractility of basal segments resembling typical Japanese octopus pot. RAO: right anterior oblique.

The main feature of TTS is the presence of transient myocardial wall motion abnormalities normally extending beyond the territory supplied by a single epicardial coronary artery [5,10]. The most common TTS morphological variant is defined as “apical ballooning” and is characterized by diffuse a- or hypokinesia of apical and midventricular myocardial segments (circumferential pattern) with basal hyperkinesia (Figure 1). Anatomical variants include “midventricular ballooning” as well as rarer forms, such as inverted or “basal” ballooning and the “focal” type [2,11]. Furthermore, right ventricular involvement (biventricular ballooning) has been reported in about 15% of TTS patients [12].

The prevalence of TTS is estimated in approximately 2%–3% of patients admitted with suspected ACS [13,14]. Individuals affected are mainly women in postmenopausal period. However, about 10% of patients are male; furthermore, a few cases affecting children have also been reported [15,16].

The pathophysiological mechanism of TTS is not yet clearly elucidated. A sympathetic overdrive seems to play a central role in TTS onset. This most likely reflects cardiac response to serum catecholamine spillover, and is precipitated in around two-thirds of cases instigated by an emotional or physical stressful trigger event [2,17]. The myocardial stunning observed in TTS has been attributed to possible coronary microcirculation dysfunction and/or the direct negative inotropic effect of catecholamine on cardiomyocytes, rather than an ischemic mechanism [18,19]. Hence, TTS was recently reclassified as “acute myocardial injury” of non-ischemic nature and excluded from the class of disorders known as myocardial infarction with non-obstructive coronary arteries (MINOCA) [20].

Due to the reversibility of myocardial wall motion abnormalities, TTS has been long regarded as a benign condition. However, recent studies show that a considerable number of patients experience severe complications, and the rate of in-hospital mortality is comparable to that of patients with ACS (1%–4.5%) [2]. The driving cause of adverse events is the acute impairment of LV function, leading to acute heart failure in 12%–45%, and to cardiogenic shock in 6%–20% of cases [21,22]. Furthermore, hemodynamic deterioration can be precipitated by LV outflow tract obstruction (LVOTO), which can be associated with systolic anterior motion (SAM) of mitral valve leaflets and moderate-to-severe mitral regurgitation [23]. While complications are mainly related to acute heart failure, a significant number of patients experience adverse events due to electrical instability. T-wave inversion with significant QT-interval prolongation are typical electrocardiographic findings in the subacute phase of TTS. Ventricular arrhythmias, such as ventricular tachycardia, torsades de pointes and ventricular fibrillation, severely complicate the in-hospital course of 2%–10% of patients [24,25]. An association between TTS and cardiac arrest has been reported in about 5% of cases [26]. Detection of intraventricular thrombi and/or cardioembolic complications has been reported in about 3% of TTS patients [27]. Further rare but life-threatening complications include ventricular septal defect and LV free wall rupture (<1%) [28,29].

## 2. Dynamic Left Ventricular Outflow Tract Obstruction in Takotsubo Syndrome

### 2.1. Left Ventricular Outflow Tract Obstruction: Definition and Clinical Features

**Figure 2:**
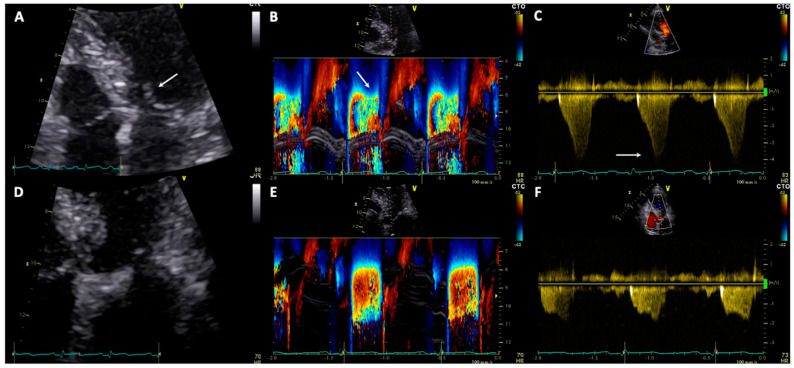
Panel (**A**) TTE with zoom on left ventricle outflow tract showing systolic anterior movement of anterior mitral leaflet during systole (see arrow); (**B**) Color M-Mode showing strong blood acceleration through left ventricle outflow tract in telesystole (see arrow); (**C**) CW-Doppler at TTE showing intraventricular peak gradient of 63 mmHg (see arrow); (**D**) TTE with zoom on left ventricle outflow tract showing absence of systolic anterior movement of anterior mitral leaflet during systole at recovery on day 6; (**E**) Color M-Mode showing regular blood acceleration through left ventricle outflow tract in telesystole at recovery; (**F**) CW-Doppler at TTE showing resolution of intraventricular gradient at recovery in the same patient. TTE: transthoracic echocardiography; CW: continuous wave.

Left ventricular outflow tract obstruction is a dynamic phenomenon defined by an intraventricular peak gradient ≥25 mmHg detected by Doppler echocardiography, and is considered hemodynamically significant in the presence of gradients ≥50 mmHg (Figure 2) [30].

The presence of LVOTO is one of the typical features of hypertrophic cardiomyopathy. Predisposing factors in this setting are asymmetrical septal hypertrophy, anterior papillary muscle displacement and elongated mitral valve leaflets [31]. Patients with LVOTO may experience symptoms such as angina, dyspnea, exertional fatigue, and syncope. Generally, LVOTO is a dynamic phenomenon and varies according to loading conditions. Reduction of preload and afterload, Valsalva maneuver, drug administration, or meals can unmask or worsen intraventricular obstruction in patients without a significant resting intraventricular gradient [32,33]. Detection of LVOTO at rest or through provocation maneuvers has important implications for risk stratification and decision making regarding medical therapy. Lifestyle measures such as avoiding dehydration and excessive alcohol intake should be advised to patients with LVOTO. Regarding medical therapy, betablockers are considered as first-line drugs in symptomatic patients. In case of contraindications to betablockers, verapamil is recommended as second-line option for symptom relief [30].

The prevalence of different causes of LVOTO has not yet been fully investigated. In a case series of 73 patients with an intraventricular pressure gradient ≥50 mmHg, besides hypertrophic cardiomyopathy (74%), other conditions such as hypertensive hypertrophy (9%), post-cardiac surgery (7%), sigmoid septum (4%), hyperkinetic LV (3%), subaortic stenosis (1.5%), and TTS (1.5%) were detected in association with LVOTO [34].

### 2.2. Pathophysiology and Prevalence of Left Ventricular Outflow Tract Obstruction in Takotsubo Syndrome

The onset of LVOTO during the acute phase of TTS is a challenging complication, and its severity varies depending on hemodynamic status. Owing to increased intraventricular afterload and systolic wall stress, leading in turn to subendocardial ischemia, LVOTO was previously considered to be the unique pathogenetic mechanism of apical TTS. However, this hypothesis lost consistency as the prevalence of LVOTO in a large series of TTS patients was reported to range between 7% and 25% [23,35,36,37]. The state of plasmatic catecholamine surge creates a paradoxical situation of mid-apical myocardial stunning and compensatory hypercontractility of the basal segments. Together with myocardial structural changes, this may create the rheological conditions for the development of dynamic obstruction. Furthermore, predisposing factors for the development of LVOTO are a small ventricular cavity and asymmetric hypertrophy of the interventricular septum (septal bulge). The onset of apical morphological variant of TTS can result in re-orientation of intraventricular drag forces, generating a “pushing” effect with SAM of mitral leaflets towards the septum. Subsequently, hypercontractility of the basal segments together with increment of septal thickness due to myocardial edema may lead to high-flow velocity in a narrowed LVOT, producing a suction effect (Venturi effect) on valve apparatus with further SAM of the anterior mitral leaflet. In addition to dynamic intraventricular obstruction, SAM may induce secondary mitral regurgitation (MR) due to leaflet malcoaptation. Furthermore, the increased afterload related to LVOTO leads in a vicious circle to a worsening of apical myocardial dysfunction owing to increased shear stress and imbalance in the oxygen supply/demand ratio. All these changes have a negative impact on cardiac output and could synergically lead to cardiogenic shock and pulmonary artery hypertension, depending on MR degree and left atrial compliance [9,38,39]. Beyond SAM, secondary MR in TTS may be due to mitral leaflet tethering as a consequence of papillary muscle displacement in patients with extended myocardial dysfunction and altered geometry of the LV. Overall, significant MR has been reported in around 20% of TTS patients and is associated with a higher rate of in-hospital adverse events. These forms of secondary MR generally resolve in the recovery of LV myocardial function and SAM-related interventricular pressure gradient resolution, respectively [23].

In a small cohort of 32 TTS patients, LVOTO was detected with echocardiography in 8 patients (25%). All patients with LVOTO showed septal bulge and SAM. Moreover, moderate-to-severe MR occurred more often in patients with TTS and LVOTO as compared to patients without LVOTO [35]. In line with these findings, De Backer et al. identified LVOTO in 6 out of 32 TTS patients (19%). In this clinical series, patients with LVOTO were older and showed septal bulge more frequently than patients without LVOTO. Furthermore, LVOTO was more often associated with SAM, significant MR, and cardiogenic shock [36]. Large national registries such as the Takotsubo Italian Network (TIN) and the Spanish Registry on Takotsubo Syndrome (RETAKO) reported a prevalence of LVOTO in TTS of 13% and 7%, respectively. In the TIN, LVOTO was associated with a composite of adverse events including acute heart failure, cardiogenic shock and in-hospital death. In the RETAKO, LVOTO was observed to be independently associated with cardiogenic shock [23,37].

In a study cohort of 44 TTS patients with LVOTO, morphological features of hypertrophic cardiomyopathy such as high interventricular septal thickness, elongated anterior mitral leaflet, and increased mitral coaptation to posterior wall distance, were detected before TTS onset. Based on these findings it has been suggested that TTS with LVOTO may represent the clinical phenotype of hypertrophic cardiomyopathy with dynamic LV obstruction triggered by a stressor [40]. However, considering that the pathophysiological mechanism of LVOTO in TTS has been previously described, this hypothesis remains solely speculative.

### 2.3. Acute Management of Patients with Takotsubo Syndrome Complicated by Left Ventricular Outflow Tract Obstruction

**Figure 3:**
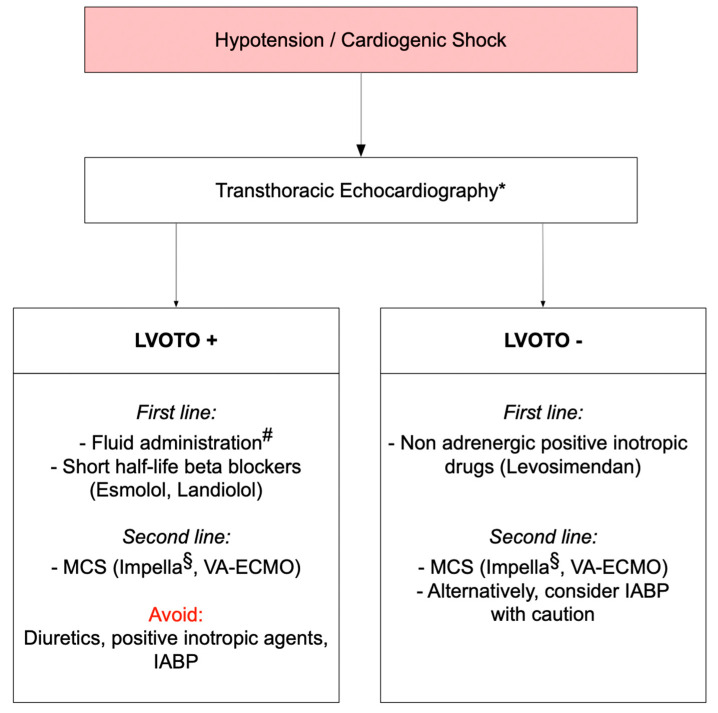
Management of patients with Takotsubo syndrome and hypotension. LVOTO: left ventricular outflow tract obstruction; MCS: mechanical circulatory support; VA-ECMO: veno-arterial extracorporeal membrane oxygenation; IABP: intra-aortic balloon pump (or intra-aortic balloon counterpulsation). * If acute coronary syndrome has not yet been ruled out, patients should undergo coronary angiography first. Slow pullback of pigtail catheter from left ventricular (LV) cavity allows early invasive assessment of intraventricular pressure gradients and can rule out LVOTO [7]. ^#^ Careful fluid administration should occur in consideration of LV ejection fraction and only in absence of acute heart failure with pulmonary oedema. ^§^ Ventricular thrombus and severe pulmonary congestion should be excluded, alternatively consider VA-ECMO.

Different mechanisms can lead to hemodynamic deterioration in patients with TTS. Prompt implementation of echocardiography is fundamental to rule out LVOTO in patients with arterial hypotension and/or hemodynamic deterioration. Early detection or rule out of LVOTO is crucial to guide patient management and avoid inappropriate therapies which could further worsen hemodynamic status (Figure 3). In patients with TTS complicated by LVOTO and cardiogenic shock, positive inotropic agents may enhance basal hypercontractility, while diuretics result in a decrease of preload with a further worsening of intraventricular pressure gradient and related MR. Therefore, positive inotropes should be discontinued, and rather than volume depletion careful fluid administration under hemodynamic monitoring should be considered [7,41].

While prognostic value of betablockers in TTS is controversial in the long term, the use of these drugs might be beneficial for management of acute heart failure until recovery of LV systolic function [2,42]. However, the safety of betablockers in the setting of hypotension and cardiogenic shock is debatable. In TTS patients with cardiogenic shock due to primary pump failure, non-adrenergic inotropic drugs such as levosimendan rather than betablockers should be considered. Conversely, beta1-selective betablockers may help decrease the intraventricular pressure gradient by reducing basal hypercontractility and improving LV filling in patients with hemodynamic deterioration due to LVOTO. Therefore, low doses of short half-life molecules such as esmolol, or alternatively an ultrashort-acting highly cardioselective β1-blocker such as landiolol, may be useful in this challenging scenario [43,44].

In critical cases with cardiogenic shock and LVOTO, mechanical circulatory support might be a valuable resource as a bridge to recovery. The Impella^®^ assist device could maintain systemic blood pressure by propelling blood from the LV into the ascending aorta, skipping the LVOTO. In addition, the position of the device in the LV outflow tract could mechanically interfere with SAM, favoring the resolution of the intraventricular gradient and related MR [45]. In selected cases with abrupt hemodynamic worsening and multiple-organ failure, veno-arterial extracorporeal membrane oxygenation may be a rescue option. The use of intra-aortic balloon counterpulsation should be avoided in patients with manifest intraventricular pressure gradient, since afterload reduction could precipitate or worsen LVOTO. In the acute phase of TTS, percutaneous mitral clip implantation could represent a rescue therapy to resolve SAM-related LVOTO and MR when unresponsive to medical therapy, especially in patients with an elongated left anterior mitral leaflet.

Due to the lack of randomized clinical trials, to date no established guidelines are available to support specific treatment options in TTS. Therefore, current recommendations solely rely on small case series and on expert opinion.

### 2.4. Late-Onset Left Ventricular Outflow Tract Obstruction in Takotsubo Syndrome: A Different Clinical Entity?

Left ventricular outflow tract obstruction has been generally described as a complication of acute phase TTS. Nevertheless, few case reports describe late-onset LVOTO occurring during or after the recovery phase. The pathogenesis of late-onset LVOTO is poorly understood. It has been hypothesized that TTS could induce permanent changes in LV geometry, affecting intraventricular septal thickness and chamber volumes. These abnormalities could remain asymptomatic in most patients, whereas they could have clinical implications, including LVOTO onset, in the presence of pre-existing subclinical myocardial disease (i.e., hypertensive cardiomyopathy) [46,47,48]. In conclusion, LVOTO can affect the clinical course of TTS not only during the acute phase, but also after the resolution of regional wall motion abnormalities. It is conceivable that late-onset LVOTO may persist over the long term and, furthermore, have prognostic implications. However, these aspects will need to be addressed in future studies.

### 2.5. Follow-Up and Long-Term Management

Long-term outcomes and management of patients with TTS and LVOTO has not yet been comprehensively investigated. Although the use of ACE inhibitors has been reported in association with better long-term outcomes in overall TTS patients, it is not known whether this beneficial effect also subsists in those with LVOTO, or whether this sub-cohort requires specific treatment [2]. Besides detecting myocardial edema in the acute or post-acute setting, CMR can reveal persistent subclinical changes, such as global microscopic fibrosis, even at long-term follow-up [49]. Therefore, this technique might be a useful tool to identify long-standing structural damages which might be associated with late-onset LVOTO or ventricular arrhythmias. In case of recurrence or persistence of symptoms, echocardiography during maneuvers such as Valsalva, standing, exercise, or postprandial should be considered to elicit or exclude late-onset LVOTO.

## 3. Conclusions and Future Perspectives

Left ventricular outflow obstruction can complicate the clinical course of a remarkable number of TTS patients, representing a dangerous liaison and one of the most challenging situations in the setting of acute cardiac care. The onset of LVOTO can lead to hemodynamic instability and cardiogenic shock, especially if in concomitance with severely reduced LV systolic function and significant MR. Beyond anatomical features such as apical ballooning and septal bulge, the identification of a specific trend of biomarkers along with the clinical and echocardiographic predictors of LVOTO might allow early recognition of a patient’s risk profile. Although the occurrence of LVOTO has been associated with a higher rate of in-hospital adverse events, the impact of LVOTO on the long-term outcome of TTS patients is still not well understood. The use of short-acting betablockers and mechanical circulatory support devices such as Impella^®^ during the acute phase of TTS complicated by LVOTO is supported by case reports and small studies but requires further testing in larger cohorts. Moreover, the long-term management of TTS patients both with acute and late-onset LVOTO is still unclear. Clinical profiles, long-term outcomes and management of critically ill patients with TTS and LVOTO remain to date poorly investigated. TTS complicated by LVOTO might be considered an emerging phenocopy categorized in the nosography spectrum of hypertrophic cardiomyopathy.
